# Phosphomannose Isomerase Is Involved in Development, Stress Responses, and Pathogenicity of Aspergillus flavus

**DOI:** 10.1128/spectrum.02027-22

**Published:** 2022-08-18

**Authors:** Sayed Usman, Chao Du, Qijian Qin, Arome Solomon Odiba, Rui He, Bin Wang, Cheng Jin, Wenxia Fang

**Affiliations:** a College of Life Science and Technology, Guangxi University, Nanning, Guangxi, China; b Guangxi Biological Sciences and Biotechnology Center, Guangxi Academy of Sciences, Nanning, Guangxi, China; c State Key Laboratory of Mycology, Institute of Microbiology, Chinese Academy of Sciences, Beijing, China; Université Côte d'Azur, CNRS, Inserm

**Keywords:** Aspergillus flavus, drug target, pathogenicity, phosphomannose isomerase, stress response

## Abstract

Aspergillus flavus causes invasive aspergillosis in immunocompromised patients and severe contamination of agriculturally important crops by producing aflatoxins. The fungal cell wall is absent in animals and is structurally different from that of plants, which makes it a potential antifungal drug target due to its essentiality for fungal survival. Mannose is one of the important components in the fungal cell wall, which requires GDP-mannose (GDP-Man) as the primary donor. Three consecutive enzymes, namely, phosphomannose isomerase (PMI), phosphomannose mutase (PMM), and GDP-mannose phosphorylase (GMPP), are required for GDP-Man biosynthesis. Thus, PMI is of prime importance in cell wall biosynthesis and also has an active role in sugar metabolism. Here, we investigated the functional role of PMI in A. flavus by generating a *pmiA*-deficient strain. The mutant required exogenous mannose to survive and exhibited reduced growth rate, impaired conidiation, early germination, disturbance in stress responses, and defects in colonization of crop seeds. Furthermore, attenuated virulence of the mutant was documented in both Caenorhabditis elegans and Galleria mellonella infection models. Our results suggested that PMI plays an important role in the development, stress responses, and pathogenicity of A. flavus and therefore could serve as a potential target for battling against infection and controlling aflatoxin contamination caused by A. flavus.

**IMPORTANCE** Aspergillus flavus is a common fungal pathogen of humans, animals, and agriculturally important crops. It causes invasive aspergillosis in humans and also produces highly carcinogenic mycotoxins in postharvest crops that threaten food safety worldwide. To alleviate or eliminate the threats posed by A. flavus, it is necessary to identify genes involved in pathogenicity and mycotoxin contamination. However, little progress has been made in this regard. Here, we focused on PMI, which is the first enzyme involved in the biosynthesis pathway of GDP-Man and thus is important for cell wall synthesis and protein glycosylation. Our study revealed that PMI is important for growth of A. flavus. It is also involved in conidiation, germination, morphogenesis, stress responses, and pathogenicity of A. flavus. Thus, PMI is a potent antifungal target to curb the threats posed by A. flavus.

## INTRODUCTION

Aspergillus flavus is one of the most common fungal pathogens of animals, plants, and humans. Being the second-most causative agent for invasive aspergillosis, A. flavus is associated with a high mortality rate, just after Aspergillus fumigatus (1, 2). During the current pandemic, COVID-19 patients have been investigated with complications of aspergillosis caused by A. flavus (3, 4). The fungus is also capable of infecting economically important crops like maize, peanut, corn, and cotton (5) and producing highly carcinogenic aflatoxins (AFs), which pose severe threats to human and animal health (6). It is of a dire need to develop new approaches and drug targets involved in pathogenicity and mycotoxin production to curb the serious threats posed by A. flavus (7).

The fungal cell wall is an important organelle providing protection to the cell, maintaining cellular integrity and proper homeostasis (8–10). Since the fungal cell wall is absent in animal cells and different from the plant cell wall, it is considered a promising target for developing antifungal drugs (11). Glucans, chitin, mannan, and glycoproteins are the main components of the fungal cell wall (12). UDP-glucose (UDP-Glc), UDP-GlcNAc, and GDP-Man are the three precursors required for cell wall biosynthesis (13). Previously, enzymes involved in the UDP-GlcNAc pathway such as GNA1, AGM1, and UAP1 have been identified as potential drug targets against A. fumigatus (14–16). Phosphomannose isomerase (PMI), phosphomannose mutase (PMM), and GDP-Man pyrophosphorylase (GMPP) are the three crucial enzymes involved in the biosynthesis of GDP-Man, which is required for cell wall mannan biosynthesis and protein glycosylation (17).

As the first committed enzyme in the GDP-Man pathway, PMI catalyzes the conversion between fructose-6-phosphate (Fru6P) and mannose-6-phosphate (Man6P), thus linking glycolysis to the GDP-Man pathway (18). This enzyme is present in mammals, bacteria, and fungi and plays an important role in cell wall synthesis, viability, and cell signaling (19). Depletion of PMI in eukaryotes results in Man6P accumulation, which leads to toxicity and glycosylation defects (20). Until now, the functional role of PMI has been reported in many organisms such as A. fumigatus, Cryptococcus neoformans, Leishmania mexicana, and Metharizum acridum (17, 21–23). The PMI deletion mutant of A. fumigatus is unable to grow without the supplementation of external mannose in the growth medium. The mutant exhibited defects in cell wall integrity, abnormal morphology, and reduced conidiation (21). Similarly, the C. neoformans PMI-disrupted mutant exhibits complete avirulence, poor capsule formation, and morphological abnormalities (17). Compared with the wild type, the L. mexicana *Δpmi* mutant exhibits attenuated virulence and was deficient in glycoconjugates synthesis, although it is able to grow in the absence of exogenous mannose (22). In the entomopathogenic fungus M. acridum, the PMI-deficient strain exhibited less virulence and also showed increased sensitivity to cell stress conditions (23). All these studies demonstrate that PMI plays a central role in development, cell wall synthesis, and virulence.

In the present study, we constructed a PMI-deficient strain of A. flavus and investigated the biological role of PMI. Our results indicated that PMI-deficient strain in A. flavus required exogenous mannose for growth. Furthermore, PMI contributed to the development, stress responses, colonization of crops, and pathogenicity of A. flavus and thus is a potential drug target for controlling the threats caused by A. flavus.

## RESULTS

### Construction of PMI deletion mutant in A. flavus.

Performing a tBLASTn search with A. fumigatus PMI (UniProt accession no. Q66WM4) revealed one *pmiA* gene in the A. flavus genome with an open reading frame (ORF) of 1,386 bp, encoding a protein of 461 amino acids (UniProt accession no. B8N4V5). A phylogenetic tree constructed using protein sequences revealed that A. flavus PMI shared 98% identity with Aspergillus oryzae and 35% identity with Fusarium oxysporum (see Fig. S1A in the supplemental material). To determine the role of PMI in A. flavus, the Δ*pmiA* mutant was constructed using homologous recombination strategy (Fig. S1B). Since *pmi* mutant of A. fumigatus requires exogenous mannose for growth (21), we used regeneration medium containing 3 mM mannose to facilitate *pmiA* mutant screening in A. flavus. The revertant strain (RT) was constructed by the insertion of *pmiA* back into the original gene locus under no mannose supplementation for screening; then, *pyrG* was inserted to replace the stop codon of the *pmiA*, thus making the RT strain autotrophic. The Δ*pmiA* and the RT strains were confirmed using four pairs of primers to ensure the correct recombination (Table S1 and Fig. S1C).

### Δ*pmiA* requires exogenous mannose for growth.

**FIG 1:**
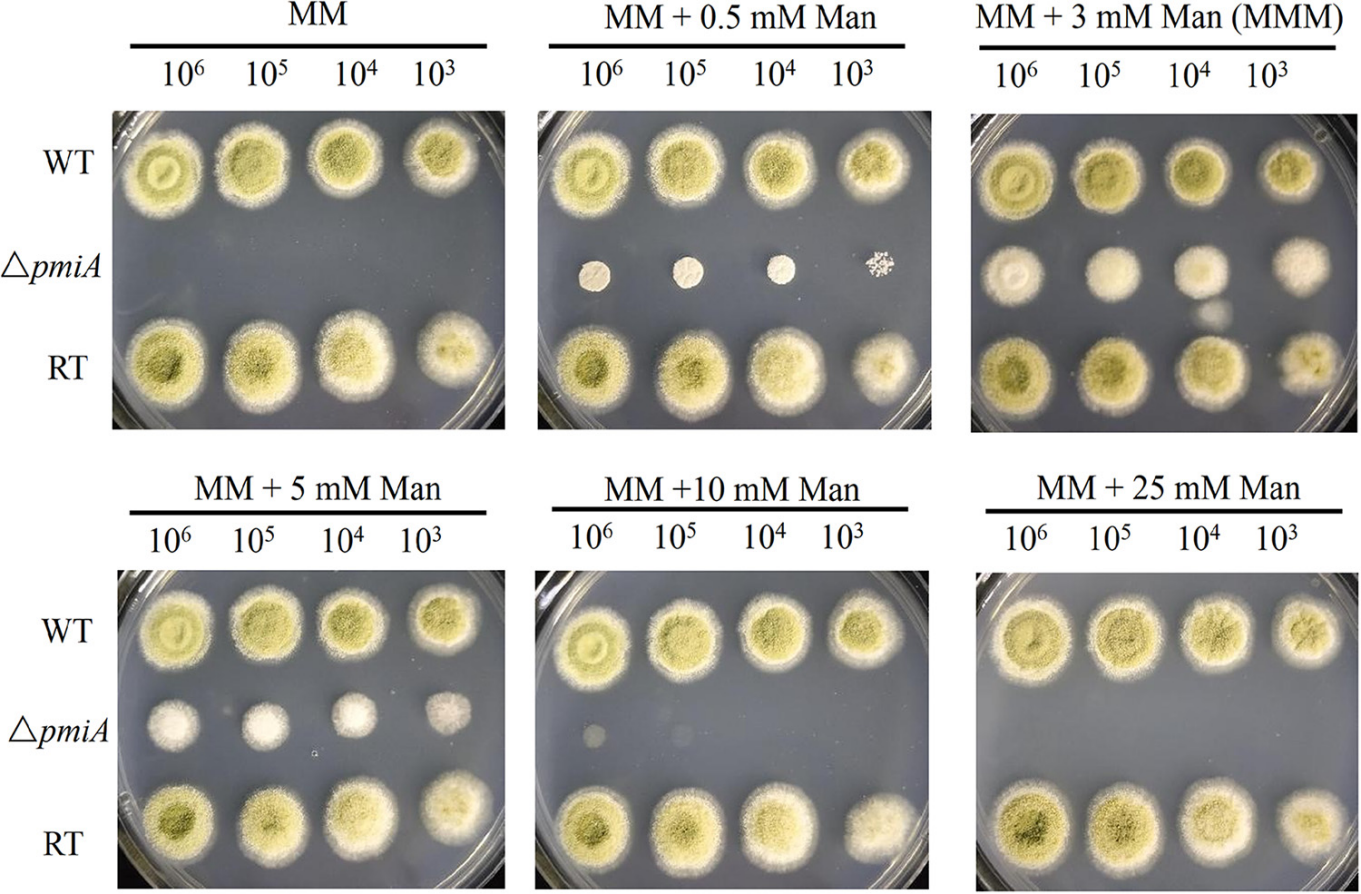
Growth of PMI mutants at different concentrations of mannose. Freshly harvested conidia of WT, *ΔpmiA*, and RT strains were serially diluted and spotted on MM media supplemented with 0, 0.5, 3, 5, 10, and 25 mM mannose. Plates were incubated at 37°C for 48 h.

In A. fumigatus, the growth of the Δ*pmiA* mutant was different in the medium supplied with different amounts of mannose. Similarly, when grown on minimal medium (MM) with various mannose (Man) concentrations (0, 0.5, 3, 5, 10, and 25 mM), the optimum growth of the Δ*pmiA* mutant was observed at 3 mM, which was defined as MMM condition for subsequent phenotypic analysis. Mannose at 0.5 and 5 mM only supported partial growth of the mutant, but over 10 mM mannose completely abolished the growth of the mutant, revealing that a large amount of mannose was not suitable for the mutant growth (Fig. 1). To further dissect this phenomenon, growth stages of the mutant in various concentrations of mannose were monitored under microscope. The Δ*pmiA* mutant displayed short ballooned germ tubes at 0.5 mM mannose, whereas longer germ tubes were seen at 3 mM and 5 mM, similar to the phenotype of the Δ*pmi1* mutant in A. fumigatus (21). Partial conidia started to germinate at 10 mM mannose after 10 h of incubation and produced ballooned hyphal tips after 48 h (Fig. S2). However, no conidia germinated at 25 mM and 40 mM mannose even after 48 h of incubation (Fig. S2). Since mannose will be phosphorylated into Man6P once it enters the cells, the above-described results implied that less than 3 mM mannose was not sufficient for the GDP-Man pathway in the mutant; higher than that amount probably led to excess Man6P accumulated due to the PMI deficiency, as well as accompanied ATP depletion for the phosphorylation process, thus keeping the conidia at the resting stage. In contrast, the wild-type (WT) and RT strains grew well irrespective of the exogenous mannose amount, indicating that apart from supplying sufficient Man6P for the GDP-Man biosynthesis pathway, the rest of the Man6P would be converted to Fru6P by PMI for energy production. Furthermore, the mutant was unable to grow on sole carbon sources, including sucrose, xylose, maltose, glycerol, galactose, mannose, GlcN, GlcNAc, and arabinose, except fructose, which only supported limited growth (Fig. S3). However, when we supplemented different concentrations of fructose (Fru) into MM containing 1% glucose, the mutant was unable to grow in all tested conditions, whereas the WT and RT strains grew well (Fig. S4). These results suggest that A. flavus PMI is important for growth and survival.

### Deletion of *pmi* led to reduced conidiation and abnormal morphogenesis.

**FIG 2:**
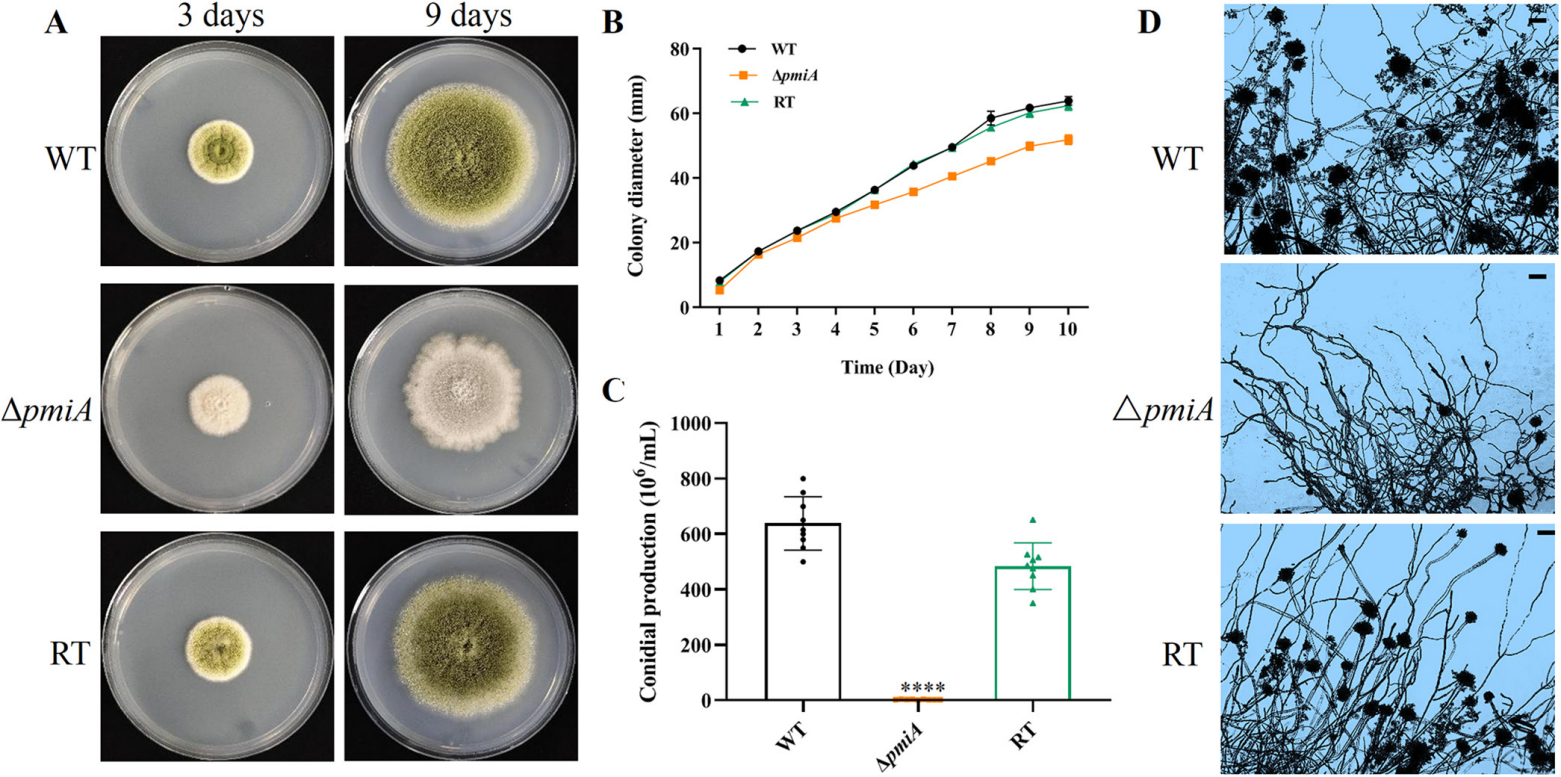
Growth of the WT, *ΔpmiA*, and RT strains on MMM. (A) Conidia of the WT, *ΔpmiA*, and RT strains were inoculated on MMM and incubated at 37°C for 3 and 9 days. (B) Colony diameter was recorded daily for 10 days for the three strains grown on MMM plates. Three replicates were performed, and data were shown as mean ± SD. (C) Conidia production was measured using a hemocytometer after 10 days of incubation on MMM at 37°C. Values represent the mean ± SD; multiple *t* test analysis was used to indicate statistical significance (****, *P* < 0.0001). (D) Conidiophore morphology of the WT, Δ*pmiA*, and RT was examined under a microscope after staining (scale bar represents 10 μm).

Since MMM is the optimum condition for the A. flavus Δ*pmiA* mutant, we further characterized its growth in more detail. When conidia of the WT, *ΔpmiA*, and RT strains were spotted on MMM, the mutant colony was less yellow than the WT and RT strains (Fig. 2A). No significant differences in the colony diameter were observed at the first 4 days, but the mutant displayed a smaller colony afterward (Fig. 2B). After 10 days of incubation, conidial counting showed that deletion of *pmiA* significantly reduced conidiation. Compared with that of the WT and RT, conidia produced by the mutant were decreased by 233- and 176-fold, respectively (Fig. 2C). To unravel the reason for the reduced conidiation in the mutant, lactic acid phenol cotton blue staining was applied and checked under a microscope. As shown in Fig. 2D, the conidiophores of the mutant were significantly less than those of the WT and RT strains, which explained the reduced conidial production.

**FIG 3:**
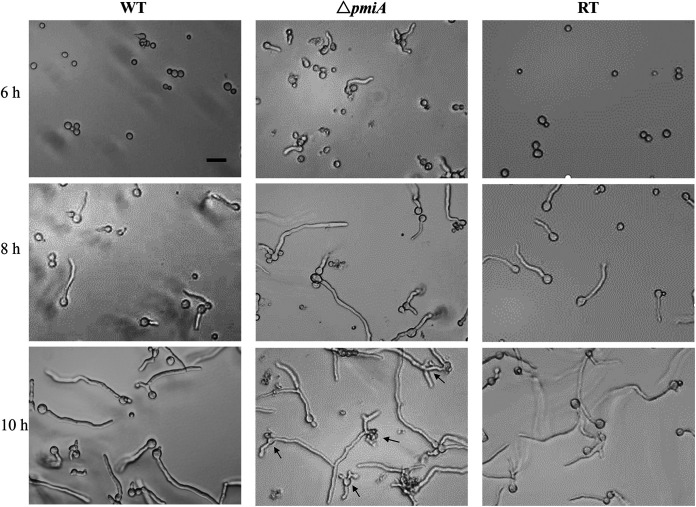
Germination morphology of the *ΔpmiA* mutant in liquid MMM. A differential interference contrast (DIC) microscope (Leica) was used to record the germination rates of the WT, *ΔpmiA*, and RT strains at 6 h, 8 h, and 10 h of cultivation at 37°C. Black arrows indicated abnormal morphology. Scale bar represents 10 μm.

In liquid MMM medium, the mutant conidia displayed faster germination, with nearly 40% of the conidia germinated at 6 h compared with 5% and 6% of the WT and RT, respectively. However, the germination rate of the mutant became slower at 8 h and 10 h than the WT and RT, and 14% of the mutant spores remained dormant even at 10 h (Table 1). Moreover, the hyphal morphology of the mutant was abnormal, including unorganized polarity or multibranches at 10 h (Fig. 3). Overall, PMI deficiency resulted in reduced conidiation, early germination, and abnormal morphogenesis in A. flavus.

**TABLE 1:** Germination rate of the Δ*pmiA* mutant in liquid MMM^*a*^

Time point (h)	Conidial germination rate (%) of:		
	WT	Δ*pmiA*	RT
6	5 ± 2	40 ± 6	6 ± 3
8	83 ± 4	81 ± 7	90 ± 1
10	94 ± 1	86 ± 1	96 ± 1

a Approximately 100 spores of each strain were captured for germination rate statistics. The experiments were repeated twice, and the data were presented as mean ± SD.

### *pmiA* deletion interrupted sclerotia formation and stress responses in A. flavus.

**FIG 4:**
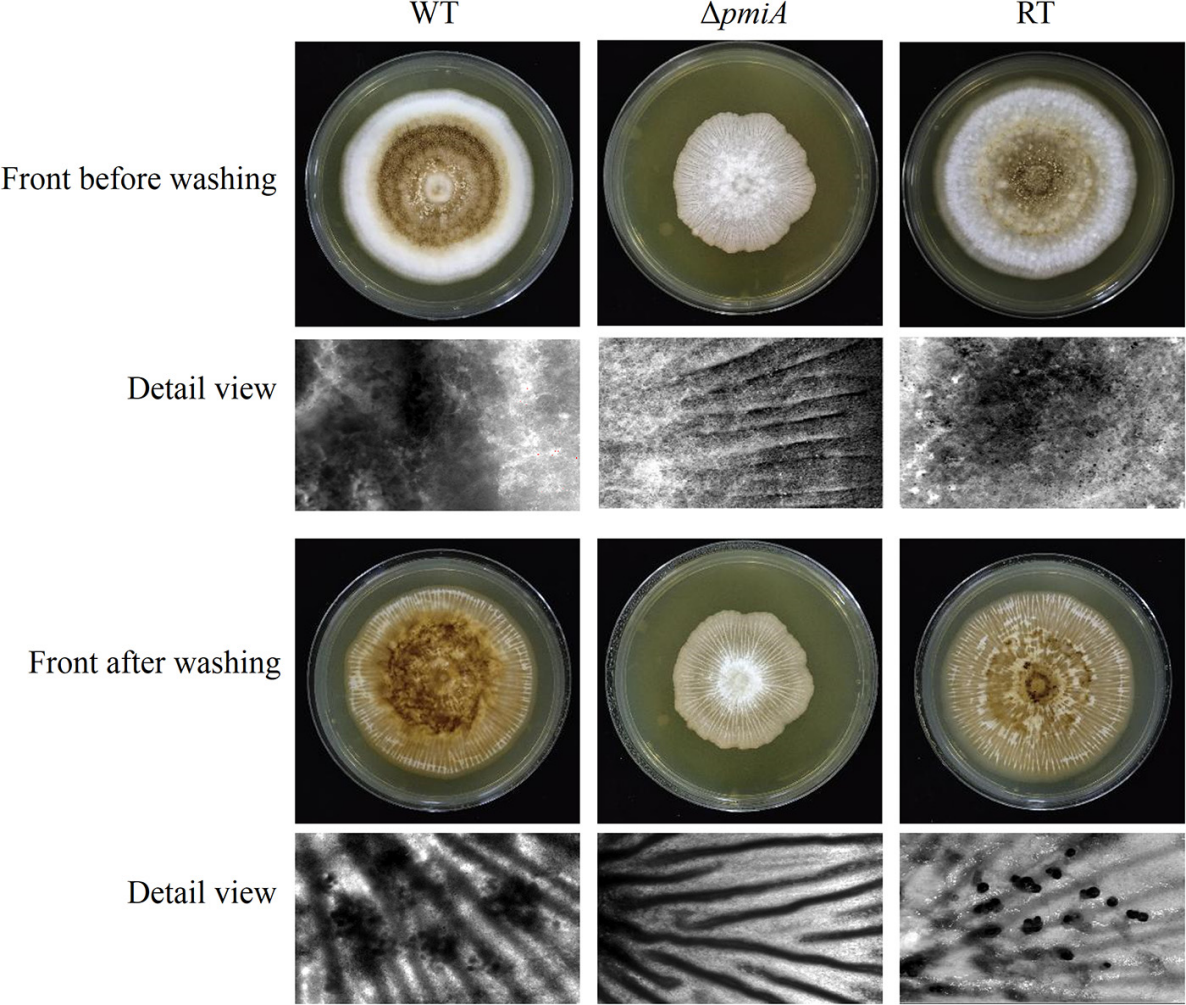
Sclerotia formation of the *ΔpmiA* mutant on YPD with 3 mM Man. Sclerotia production before and after washing with ethanol was viewed under a stereomicroscope after 10 days of incubation at 37°C in the dark.

To overcome the stresses of the external environment, A. flavus often develops sclerotia, which act as repositories for the production of sexual spores (24). To evaluate the role of PMI in sclerotia formation, conidia of the WT, Δ*pmiA*, and RT strains were spotted in yeast extract-peptone-dextrose (YPD) medium with 3 mM mannose supplementation. After incubation at 37°C for 10 days, the spores were washed off with 75% ethanol. As shown in Fig. 4, the formation of sclerotia was severely impaired in the *ΔpmiA* mutant in contrast to that in the WT and RT, indicating that PMI might be involved in withstanding adverse environmental conditions. We next tested the responses of the mutant toward chemical and osmotic stresses.

**FIG 5:**
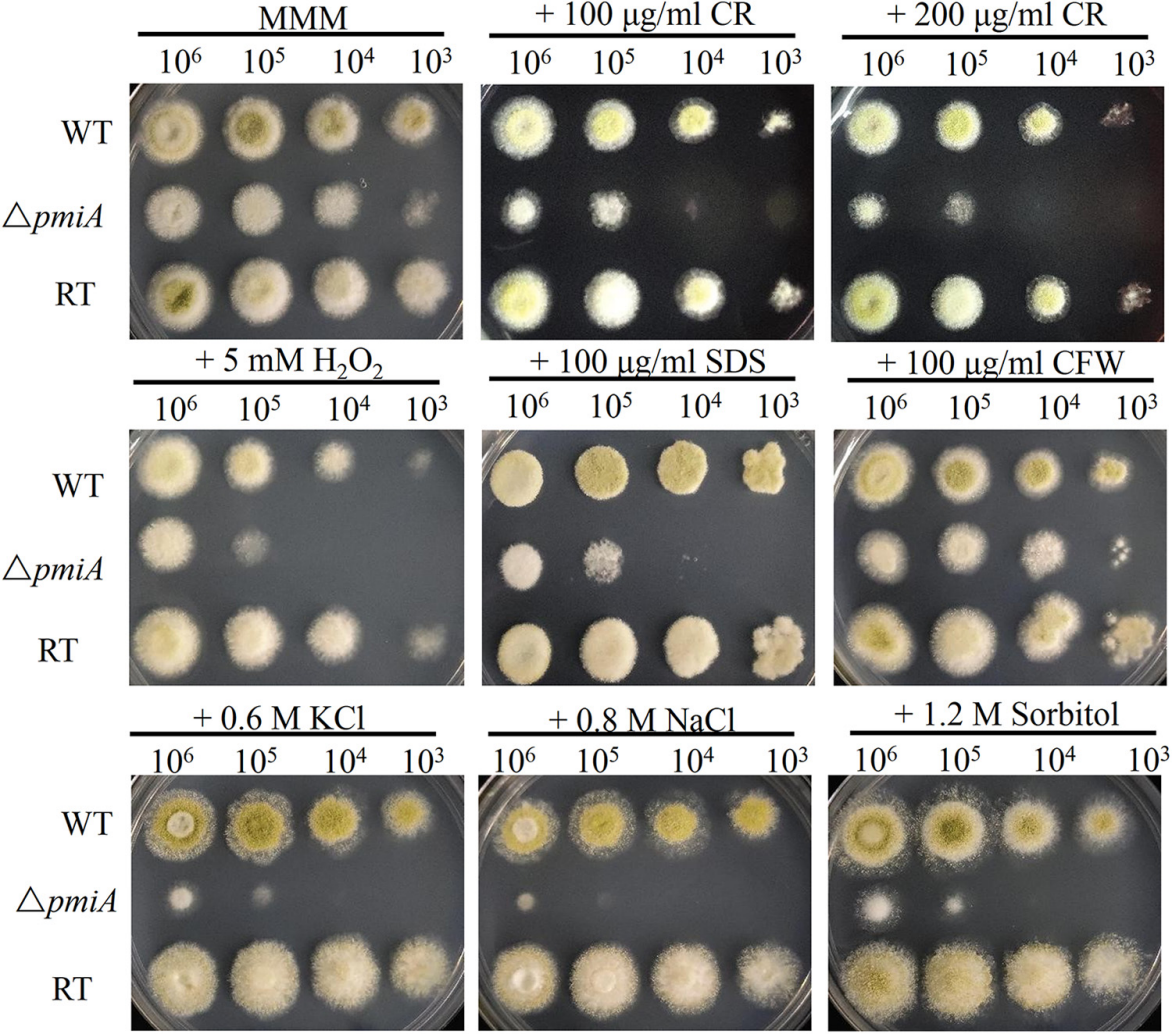
Responses of the Δ*pmiA* mutant to various stresses. We inoculated 10^6^ to 10^3^ serially diluted conidia on MMM supplemented with 100 μg/mL and 200 μg/mL CR, 5 mM H_2_O_2_, 100 μg/mL SDS, 100 μg/mL CFW, 0.6 M KCl, 0.8 M NaCl, and 1.2 M sorbitol. Plates were incubated at 37°C for 2 days.

The results indicated that the cell wall stressor Congo red (CR), cell membrane stressor sodium dodecyl sulfate (SDS), and the oxidative stressor H_2_O_2_ significantly inhibited the growth of the *ΔpmiA* mutant but not WT and RT strains (Fig. 5). We further quantified cell wall components by using a previously reported method (25). Our results demonstrated that the amounts of cell wall polysaccharides were not significantly changed in the Δ*pmiA* mutant compared with the WT and RT strains (Fig. S5). However, lack of PMI might contribute to defective mannosylation of cell wall proteins, leading to increased sensitivity to CR and SDS. It has been reported that osmotic stabilizers could rescue fungi conidiation (26). Unexpectedly, the mutant displayed supersusceptibility to all osmotic stabilizers, indicating that those osmotic stabilizers exert stresses on the mutant (Fig. 5). It is obvious that *pmi* deletion interrupted sclerotia formation and stress responses in A. flavus.

### *pmi* deletion affected colonization in crop seeds.

**FIG 6:**
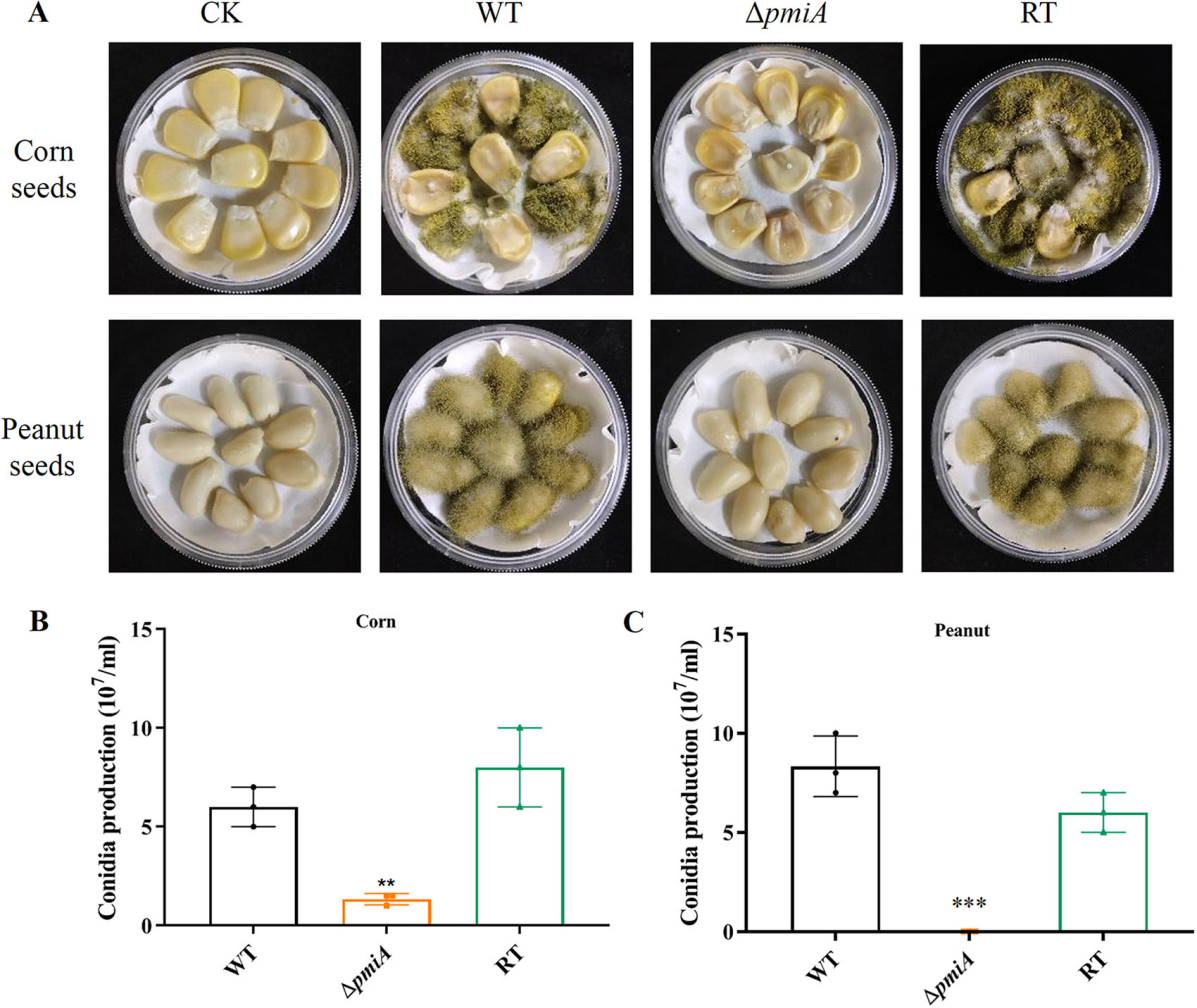
Colonization assay of A. flavus strains on crop seeds. (A) We inoculated 10^6^ conidia from the WT, *ΔpmiA*, and RT strains on corn and peanut seeds, and the plates were incubated at 28°C for 6 days in the dark. (B) Conidia washed from infected peanut seeds were counted by hemocytometer. (C) Conidia washed from infected corn seeds were counted by hemocytometer. The experiment was conducted in three biological repeats.

Since the above-described results indicated that PMI affected growth, conidiation, and sclerotia formation in A. flavus, we therefore hypothesized that the pathogenicity of the Δ*pmiA* mutant might be deficient in plant seeds. To test the hypothesis, peanut and corn seeds were used for infection. After incubation at 28°C for 6 days in the dark, peanut and corn seeds were severely infected by the WT and RT, with a large number of conidia produced on the surface of the seeds, whereas the seeds infected by the mutant displayed no symptoms (Fig. 6A). The conidia washed from the seeds infected by the mutant were significantly less than those from the seeds infected by WT and RT (Fig. 6B and C). To further assess the cause of reduced pathogenicity of the Δ*pmiA* mutant, we tested the growth rate of the mutant on agar plates supplemented with 1%, 3%, and 5% of peanut or corn powder as nutrients. As shown in Fig. S6, the mutant conidium was unable to germinate on those plates. Additionally, the mycelium of the mutant was also unable to grow on the above-described plates (Fig. S7), suggesting that the Δ*pmiA* mutant cannot utilize crop seed nutrients for growth.

**FIG 7:**
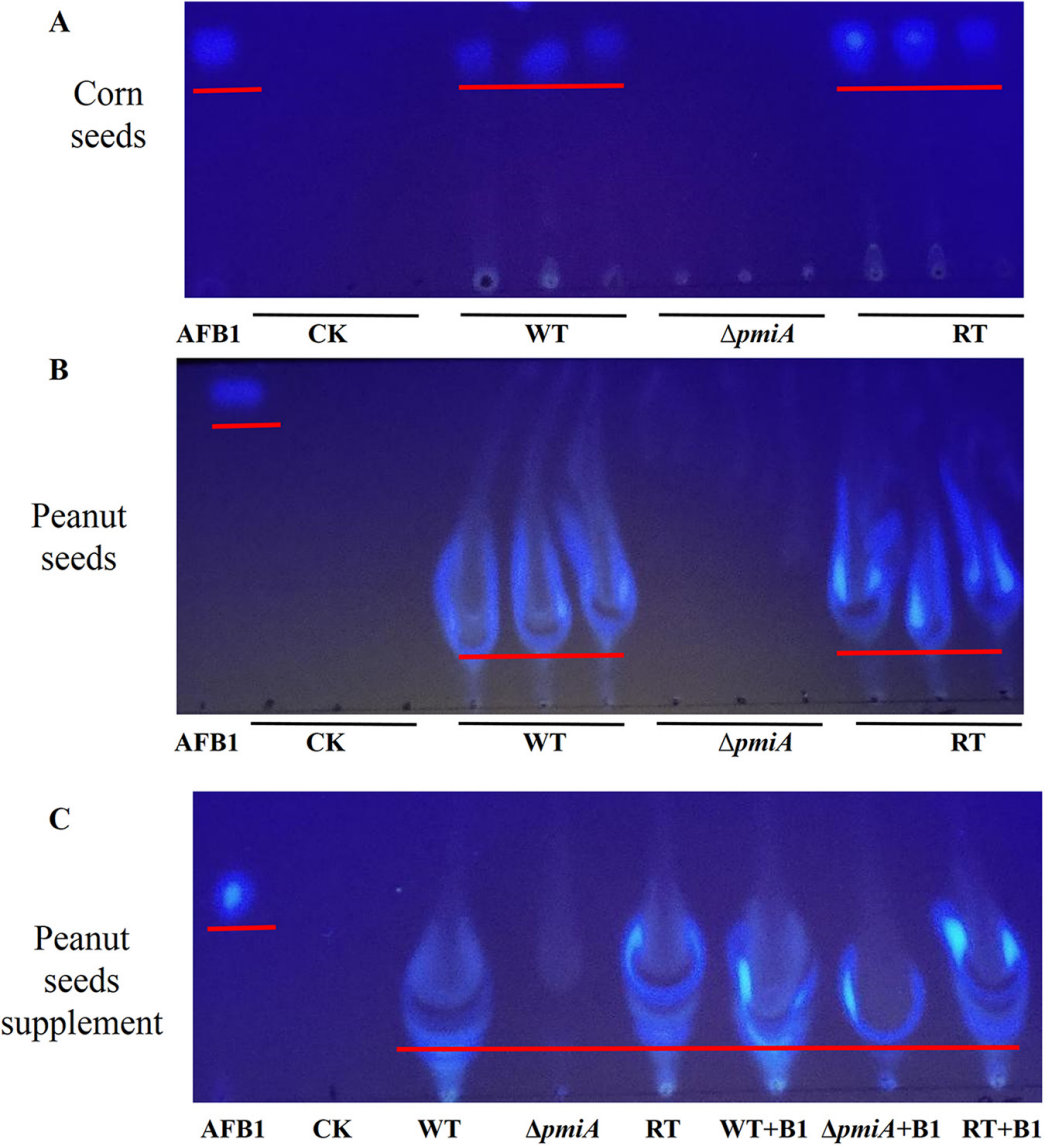
TLC analysis of AFB1 produced in infected seeds. Aflatoxin was extracted from conidia washed from infected peanut and corn seeds with an equal amount of chloroform. Chloroform was used as a control (CK). (A) Detection of AFB1 in corn seeds; (B) detection of AFB1 in peanut seeds; (C) verification of peanut oil interference on TLC. AFB1 standard was added into each sample for comparison.

We further analyzed the amount of aflatoxin B1 (AFB1) accumulated in the infected seeds. Thin-layer chromatography (TLC) analysis revealed that AFB1 was accumulated in the seeds infected by the WT and RT strains, whereas no AFB1 was detected in the seeds infected by the Δ*pmiA* mutant (Fig. 7). These results demonstrate that the Δ*pmiA* mutant loses its ability to colonize and grow on crop seeds, and AFB1 cannot be accumulated in the mutant infected crops.

**FIG 8:**
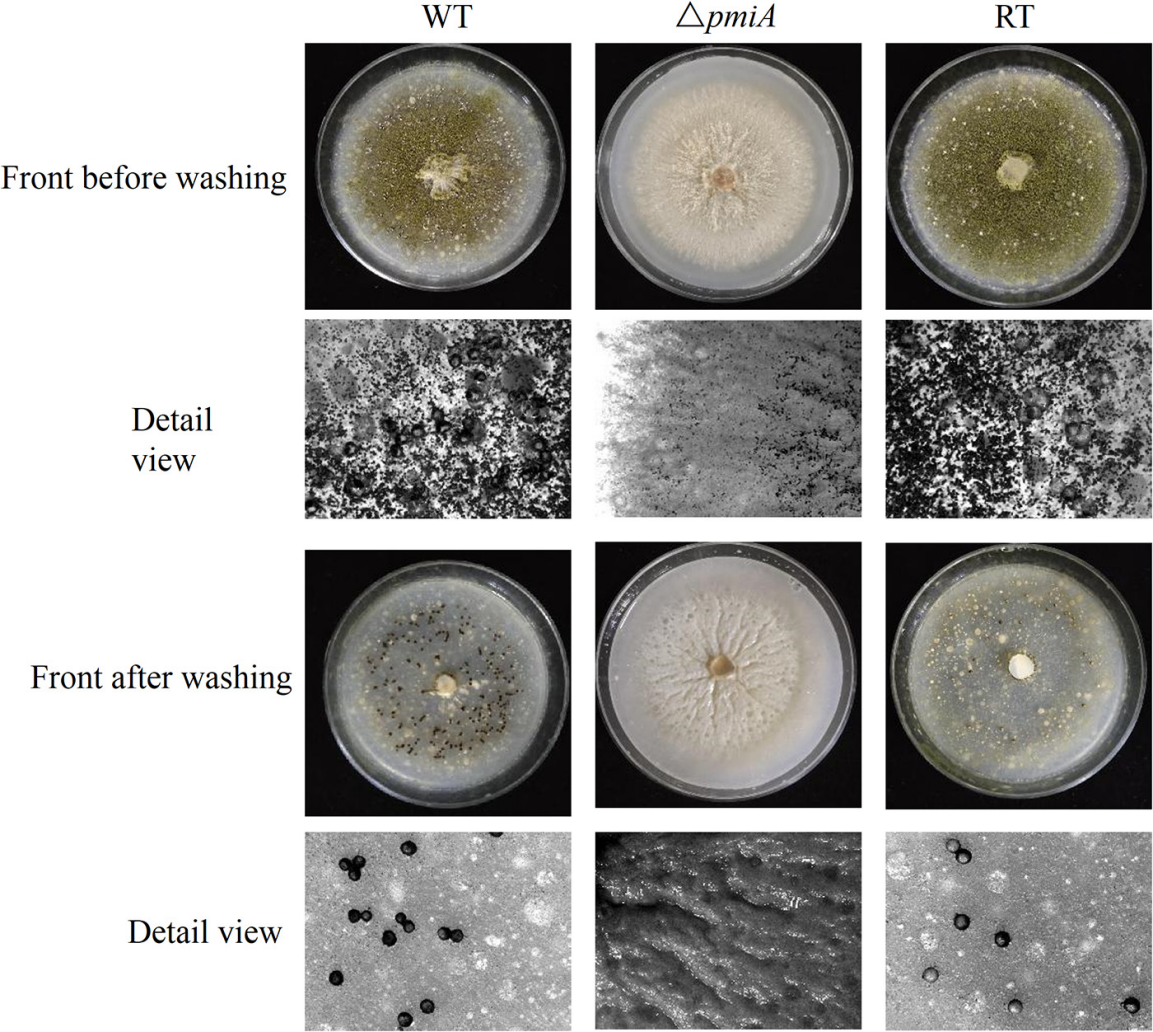
Sclerotia formation of the *ΔpmiA* mutant on MMM supplemented with 1% peanut powder. Sclerotia production before and after washing with ethanol was viewed under a stereomicroscope after 7 days of incubation at 28°C in dark. We applied 1% peanut powder plates for the WT and RT strains.

Since the above-described plant infection studies were carried out at 28°C, we characterized the temperature effect on the growth of the mutant. As shown in Fig. S8A, in contrast to the WT and RT strains, the mutant displayed similar growth patterns at 28°C, 37°C, and 42°C. Furthermore, the mutant exhibited reduced conidiation and retarded growth rate at 28°C (Fig. S8B through D), similar to the performance at 37°C. When we used YPD plates supplemented with mannose for sclerotia production at 28°C, the WT and RT strains showed less sclerotial structures; therefore, the peanut powder plates (1%) were applied for the WT and RT strains. MMM medium was added to support the growth of mutant. After 7 days incubation in dark, both the WT and RT produced clear sclerotia, whereas no sclerotia were produced by the mutant (Fig. 8). Since PMIs are not important/essential in plants and are reported absent in many plants (27), it is promising that inhibitors targeting A. flavus *pmiA* will be applicable in the agricultural field, at least for prophylaxis of postharvest crop contamination.

### PMI deficiency led to attenuated virulence in animal infection models.

**FIG 9:**
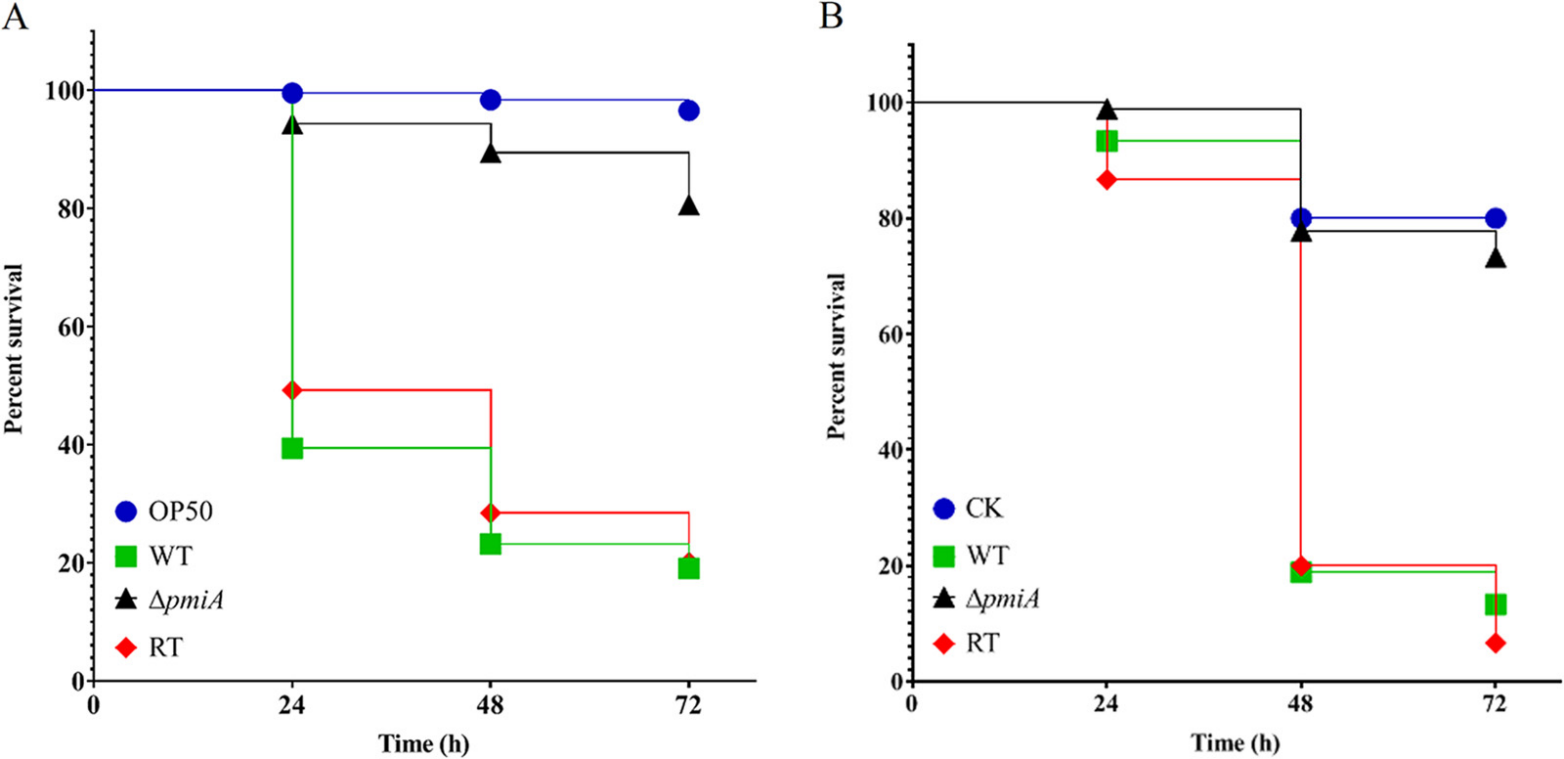
Virulence tests of A. flavus strains in C. elegans and G. mellonella models. (A) Kaplan-Meier survival plot of *glp-4* (bn2) and *sek-1* (km4) worms fed by the conidia of indicated strains after 16 h of infection. Worms infected with Escherichia coli OP50 strain were used as control. For each strain, three biological repeats (each with triplicates) were conducted. (B) Kaplan-Meier survival plot of G. mellonella larvae at 24, 48, and 72 h after infection with conidia of the indicated strain. Larvae treated with heat-killed spores were used as control (CK). The incubation temperature was 28°C. For each strain, three biological repeats (each with triplicates) were conducted.

Since A. flavus is the second causative agent of aspergillosis and a serious threat to humans, we next investigated the pathogenicity of the Δ*pmiA* mutant in a Caenorhabditis elegans infection model (28). The survival rates of worms infected with the WT, mutant, and RT strains were counted at intervals of every 24 h and then plotted with Kaplan-Meier survival curves. As shown in Fig. 9A and Table S2A, the *ΔpmiA* mutant was found to be significantly less virulent than the WT and RT strains. After 72 h of infection, the survival rates of the worms infected by the WT, mutant, and RT were 20%, 79%, and 19%, respectively. Since hyphal formation is critical for virulence (29), the hyphal filamentation rates in infected worms were also recorded. Similar to the virulence results, there was no hyphal filamentation in the *ΔpmiA* mutant, whereas the WT and RT-infected worms displayed 55% and 51% filamentation, respectively (Table S2A).

The pathogenicity was also evaluated in a Galleria mellonella infection model at 28°C, and a similar trend was observed as in the C. elegans model. The larvae infected with the *ΔpmiA* mutant and heat-killed conidia showed 73% and 80% survival rates, respectively, after 72 h of infection compared with those of the WT and RT strains (13% and 7%, respectively) (Fig. 9B and Table S2B). Collectively, these results indicate that PMI is involved in the pathogenicity of A. flavus in both C. elegans and G. mellonella infection models.

## DISCUSSION

As an opportunistic fungal pathogen, A. flavus ranks second after A. fumigatus in causing deadly invasive aspergillosis (2, 30). Moreover, A. flavus is capable of producing aflatoxins in maize, groundnut, cassava, almond, peanuts, and many other important agricultural crops, which is a major concern for food safety (31–33). Among aflatoxins, AFB1 is highly carcinogenic and causes severe digestive, nervous, vascular, and estrogenic defects, thus posing threats to animals and humans (34, 35).

The fungal cell wall maintains the structure and integrity of the fungal cell (36) and thus is associated with physiology and essential biological processes (37). Since the fungal cell wall structure is unique to fungi, different from plants’ cell walls and absent in human cells, it is proposed as a promising target for antifungal strategies (38). Mannose is an essential component for the synthesis of cell wall mannoproteins and mannan (13). As a primary mannose donor, GDP-Man is synthesized from Fru6P by three bioactive enzymes, PMI, PMM, and GMPP. Fru6P is also an important intermediate in glycolysis (39). The function of PMI has been characterized in A. fumigatus, revealing its essentiality for cell wall synthesis and morphogenesis (21). In the current study, the functional role of PMI in A. flavus was investigated by constructing a deletion mutant and conducting phenotypic analysis.

Similar to the A. fumigatus Δ*pmi* mutant, the PMI-deficient strain in A. flavus was unable to grow without the supplementation of exogenous mannose. Three millimolar mannose was sufficient for the optimal growth of the mutant whereas, higher concentrations of mannose (>10 mM) inhibited the growth of the mutant (Fig. 1 and see Fig. S2 in the supplemental material). On the other hand, the WT and RT grew well irrespective of exogenous mannose. These results indicate that PMI is required for balancing and buffering mannose metabolism in A. flavus. The high mannose inhibition on the germination and growth of the mutant was likely due to the phosphorylation and dephosphorylation of mannose accompanied by intracellular ATP depletion, just like the “honeybee effect” (40). We also noticed that fructose as the sole carbon source could restore partial growth of the mutant, whereas when it was added into MM in which glucose was the main carbon source, the mutant exhibited no growth at all (Fig. S3 and S4). Since the mutant required as little as 0.5 mM mannose to grow, we speculate mannose is an unavoidable impurity (<1%) during fructose production, thus supporting partial growth of the mutant when fructose was the sole carbon source. However, when both glucose and fructose were supplied, the uptake of fructose (and its impurities) was blocked due to the carbon catabolite repression (41); therefore, the growth of the mutant could not be restored. Both small and large amounts of mannose severely impaired the germination of the Δ*pmiA* mutant in which low concentrations of mannose led to abnormal hyphal morphology, whereas high concentrations of mannose blocked the germination of the Δ*pmiA* mutant (Fig. 3 and Fig. S2).

PMM and GMPP in the GDP-Man biosynthesis pathway have been shown to have an impact on the development of A. fumigatus. It has been reported that PMM in A. fumigatus is indispensable for survival and its deletion leads to altered cell wall organization and certain morphological abnormalities such as reduced conidiation, abnormal polarity, retarded growth, and germination (42). Similarly, the GMPP conditional mutant of A. fumigatus exhibited phenotypic defects, including hyphal lysis, rapid germination, reduced conidiation, and altered cell wall integrity (43). Consistently, our study showed that A. flavus PMI was involved in growth, conidiation, and morphogenesis (Fig. 2 and 3). Despite that functions of PMIs in closely related A. fumigatus and A. nidulans have been studied previously, our work that focused on the A. flavus Δ*pmiA* mutant revealed different phenotypes, including faster germination, interrupted sclerotia formation, and supersusceptibility to osmotic stabilizers, which usually act for salvage competence in other fungal PMI mutants (21, 44).

Since PMI is the first committed enzyme in the GDP-Man biosynthesis pathway, we proposed that PMI deletion may have direct effects on the cell wall integrity. Indeed, the A. flavus *ΔpmiA* mutant was sensitive to CR, but cell wall polysaccharides of the mutant were similar to those of the WT and RT strains (Fig. 5 and Fig. S5), implying that disturbances in cell wall integrity might be due to defective glycosylation/mannosylation of cell wall components. Furthermore, the hypersensitivity of the mutant to SDS suggested a marked defect in cell membrane structure. Our results also demonstrated that the *pmi*-deficient strain did not develop sclerotia either at 28°C (Fig. 8) or 37°C (Fig. 4), which are repositories for the production of sexual spores in harsh and stress conditions (24). This might be the reason for the reduced tolerance of the mutant toward stresses, including SDS, H_2_O_2_, and osmotic stabilizers (Fig. 5).

Infection of plants by pathogenic fungi is a complex process (45). Our results from the infection assays demonstrated that the *ΔpmiA* mutant was unable to colonize and grow on corn and peanut seeds (Fig. 6A to C) and thus was unable to produce AFB1 (Fig. 7). Similarly, the *pmi*-deficient strain was inefficient in causing virulence in C. elegans and G. mellonella models (Fig. 9). All these results imply that A. flavus PMI has a critical role in triggering virulence in animals and plants.

In conclusion, our study here fully characterized the function of PMI in A. flavus, revealing that PMI in A. flavus is essential for growth, conidiation, stress responses, and pathogenicity in both plant and animal models. Despite that developing an inhibitor selectively targeting A. flavus PMI but not a human PMI orthologue is far away, newly rising approaches, such as fragment-based drug discovery, will facilitate the exploration of selective inhibitors against highly conserved potent targets (46). Nonconserved sites of the target could be explored for selectivity, such as the selective inhibitors against Plasmodium falciparum *N*-myristoyltransferase (NMT) that were developed based only on the single-residue difference from human orthologue (47, 48). Recently, we also confirmed that A. fumigatus phosphoglucose isomerase (PGI) possesses an exploitable site and space for developing selective inhibitors (49). Applying PMI inhibitors in the agricultural field will be more reachable since the PMIs are less ubiquitous in the kingdom Plantae and are reported absent in many plants (27). Therefore, PMI could be a promising target to battle against infection and control crop contaminations caused by A. flavus.

## MATERIALS AND METHODS

### Fungal strains, media, and culture conditions.

The uracil auxotroph strain CA14*Δku70ΔpyrG* was used as the parental strain for transformation, and CA14*Δku70* was used as the wild-type (WT) strain for phenotypic analysis. All strains were cultured at 37°C or 28°C and stored at 4°C for the short term and at −80°C in glycerol stocks for the long term. MM medium was used for the WT and revertant strains. MM supplemented with 5 mM uracil and uridine or with 3 mM mannose was used for the *pyrG* auxotroph and mutant strains, respectively. The primers used in the study are listed in Table S1 in the supplemental material.

### Phylogenetic analysis.

The PMI sequence for A. flavus was downloaded after a tBLASTn search of the genome using the A. fumigatus PMI sequence. PMI sequences from Aspergillus caelatus, Aspergillus candidus, Saccharomyces cerevisiae, Aspergillus pseudotamarii, Aspergillus pseudonomiae, Aspergillus bombycis, Aspergillus nomius, Aspergillus oryzae, Aspergillus fumigatus, Fusarium oxysporum, and Candida albicans were obtained from the National Center of Biotechnological Information (NCBI). The phylogenetic tree was produced using the neighbor-joining method and bootstrap test of 1,000 replicates in MEGA 5.0 software (50).

### Construction of mutant and revertant strains.

The mutant and revertant strains were constructed by homologous recombination strategy. Upstream and downstream flanking regions of 1.5 kb each from CA14*Δku70* and A. fumigatus *pyrG* selectable marker from the *AfpyrG* plasmid were PCR amplified using PrimeStar HS TaKaRa Taq mix (code no. R010Q). The three fragments were assembled in pCE zero vector using the seamless cloning kit (Vazyme, China). Then, the fused fragment, *up-pyrG-down*, was amplified from the vector and transformed into the CA14*Δku70ΔpyrG* protoplast to generate the *ΔpmiA* mutants by screening on MM medium supplemented with mannose. The revertant strain (RT) was constructed according to the method previously described (51). In the first round, the 4.5-kb flanking region from upstream to downstream of the WT genomic DNA was amplified and transformed into mutant protoplasts to screen *pyrG* auxotrophic strains on transforming plates without mannose supplementation. In the second round, the *pyrG* marker was inserted between the gene and downstream region and transformed to the screened *pyrG* auxotrophic protoplasts. All the strains were confirmed by PCR using four pairs of primers to ensure homologous recombination.

### Effect of exogenous mannose on the strains.

To optimize the growth condition of the mutant, MM plates with different concentrations of mannose (0, 0.5, 3, 5, 10, and 25 mM) were prepared. Serially diluted conidia (10^6^ to 10^3^) from WT, Δ*pmiA*, and RT strains were spotted on these plates and were incubated at 37°C for 2 days.

### Growth phenotype, conidia production, and sclerotium formation analysis.

To test the growth rate on solid plates, 10^6^ conidia from the WT, Δ*pmiA*, and RT strains were inoculated onto the center of MMM solid medium plates for incubation at 37°C or 28°C. Colony diameters for each strain were recorded every 24 h along the same line for 10 days. Colony morphologies were photographed on the 3rd and 9th days of inoculation, and growth curves were drawn after 10 days. Conidia were collected by washing with 0.2% (vol/vol) Tween 20 and were counted by hemocytometer.

To analyze sclerotia production, 10^6^ conidia from the WT, Δ*pmiA*, and RT strains were inoculated onto the center of sclerotial induction medium YPD containing 1% glucose and 3 mM mannose as carbon sources. Plates were incubated at 37°C in the dark to induce sclerotia. Colony morphology was photographed under a microscope after 10 days of incubation. The mycelia and spores on the plates were washed out with 75% alcohol, and the colony morphology was again photographed under a microscope. In addition, the sclerotia production of WT and RT strains at 28°C was performed on 1% peanut powder plates. Sclerotia formation of the *ΔpmiA* mutant at 28°C was conducted on MMM supplemented with 1% peanut powder.

To observe the morphology of conidiophores, a small steel ring with holes was fixed between a slide and a coverslip with wax solution. The holes were sealed with wax by injecting molten MMM medium into the ring. Conidia from WT, Δ*pmiA*, and RT strains were inoculated to the solidified medium by a fine wire dip. The samples were incubated at 37°C in the upright position and were examined every 24 h. When conidiophore growth was observed, a solution of lactic acid phenol cotton blue dye was added on the conidiophore cluster with a syringe, and photos were taken under a microscope.

### Hyphal morphology and germination statistics.

Spore germination experiments were performed in 96-well plates. Conidia of 10^5^ were inoculated into 200 μL MMM liquid medium and incubated at 37°C. The spore germination status was observed under a microscope after 6 h, 8 h, and 10 h of incubation. The hyphal morphology was randomly photographed, and over 100 cells of each strain were selected to count the germination rate. The mycelial morphology of the mutant was also observed under different mannose concentrations (0.5, 3, 5, 10, 25, and 40 mM) and was photographed at 10 h and 48 h.

### Assays for sensitivity to stress and temperature conditions.

Stresses, including Congo red (CR), calcofluor white (CFW), sodium dodecyl sulfate (SDS), sodium chloride (NaCl), potassium chloride (KCl), sorbitol, and hydrogen peroxide (H_2_O_2_), were conducted. Briefly, serially diluted conidia (10^6^ to 10^3^) from the WT, Δ*pmiA*, and RT strains were point inoculated on MMM plates containing the mentioned stress chemicals at different concentrations and incubated at 37°C.

### Cell wall content analysis.

The cell wall components were determined using the previously described method (25). Briefly, 10^7^ conidia of the WT, Δ*pmiA*, and RT strains were inoculated into MMM liquid medium and cultured at 37°C, 200 rpm. After 48 h, mycelia were harvested by filtration and were converted to fine powder by grinding in liquid nitrogen. SDS-BME [50 mM Tris, 50 mM EDTA, 2% SDS, and 1 mM Tris(2-carboxyethyl)phosphine hydrochloride (TCEP)] was added to the powder and boiled at 100°C for 40 min. After centrifugation, cell wall fractions were thoroughly washed with Milli-Q water and were freeze-dried for 3 days. We added 75 μL of 72% H_2_SO_4_ to 10 mg cell dry mass and left for 3 h at room temperature. The pellet was then resuspended in 0.95 mL Milli-Q water and boiled at 100°C for 4 h. After neutralization to pH 7 by Ba(OH)_2_, the samples were left at 4°C overnight. The monosaccharide content in the supernatant was determined by high-performance anion-exchange chromatography coupled with a pulsed amperometric detector (HPAEC-PAD) using a CarboPac PA-10 anion exchange column equipped with an Amino trap guard column at room temperature. Elution was performed at a flow rate of 1 mL min^−1^ using 18 mM NaOH at room temperature.

### Peanut and corn seed infection.

Fresh peanut and corn seeds of similar size and shape were selected and washed with 0.05% sodium hypochlorite for 3 min and then 75% ethanol for 1 min. To avoid germination, the endosperm was removed by toothpicks. The seeds were then washed three times with sterile water; inoculated with 10^6^ conidia of WT, Δ*pmiA*, and RT strains; and incubated at 28°C for 6 days in the dark. During the incubation period, the humid conditions for seeds were maintained by using wet filter paper. After incubation, the infected seeds were transferred to 20 mL of 0.2% Tween 20 in 50-mL tubes. The tubes were shaken at 200 rpm for 5 min, and the conidia were released. One-milliliter aliquots of conidia were taken from each sample, serially diluted, and counted using a hemocytometer. To check the growth ability of the mutant under a natural nutritional environment, peanut and corn seeds were ground to powder form. Concentrations of 1%, 3%, and 5% (wt/vol) corn and peanut powder were mixed with agar to prepare solid plates. Fresh conidia (10^5)^ of the WT, Δ*pmiA*, and RT strains were inoculated in the center of the plates and incubated at 37°C for 6 days. In addition, the mycelium slices of the WT, Δ*pmiA*, and RT strains were placed onto the center of the above-mentioned peanut and corn plates and cultured at 28°C.

### TLC and detection of AFB1.

Using an equal volume of chloroform, aflatoxin was extracted from 500 μL of filtrate, and the chloroform layer was transferred to a fresh tube, which was then dried at 70°C. Aflatoxin production was detected by TLC using a solvent system of chloroform/acetone at 9:1 and visualized under UV at 365 nm wavelength.

### Virulence studies in animal models.

In accordance with an established virulence test using the Caenorhabditis elegans-based infection model (28), we tested the pathogenicity of the WT, *ΔpmiA*, and RT strains. Briefly, worms were infected with 10^8^ conidia of the fungal strains at 25°C. After 16 h, the preinfected worms were transferred to killing assay medium (brain heart infusion [BHI+]) at 20°C. Survival rates of worms at 24 h, 48 h, and 72 h postinfection were recorded. The hyphal filamentation rate represents the rate of hyphal filaments that protruded from worm bodies.

For virulence test in Galleria mellonella, six instar larvae were selected and divided into 4 groups, each containing 90 larvae. Conidia of 10^4^ from WT, *ΔpmiA*, and RT strains were injected into the hind proleg of larvae by Hamilton syringe. Larvae treated with heat-killed spores were used as a control (CK). After infection, the larvae were incubated at 28°C, and the survival rate was recorded for each group at 24 h, 48 h, and 72 h. Larvae that had apparent melanization or dark spots and were unable to move were considered dead.

### Statistical analysis.

GraphPad Prism 8 software was used to plot all the curves in this study. All data were presented as mean ± standard deviation (SD). The comparative analysis of statistical significance was done by one-way analysis of variance (ANOVA) for multiple-comparison analysis.
