# Supplementary material for: Phosphomannose Isomerase Is Involved in Development, Stress Responses, and Pathogenicity of Aspergillus flavus
Source: Microbiol Spectr. 2022 Aug 18;10(5):e02027-22. doi: 10.1128/spectrum.02027-22 (PMC9603912; doi:10.1128/spectrum.02027-22)
Supplement: Supplemental file 1 — Fig. S1 to S8 and Tables S1 and S2. Download spectrum.02027-22-s0001.pdf, PDF file, 1.3 MB [file spectrum.02027-22-s0001.pdf]

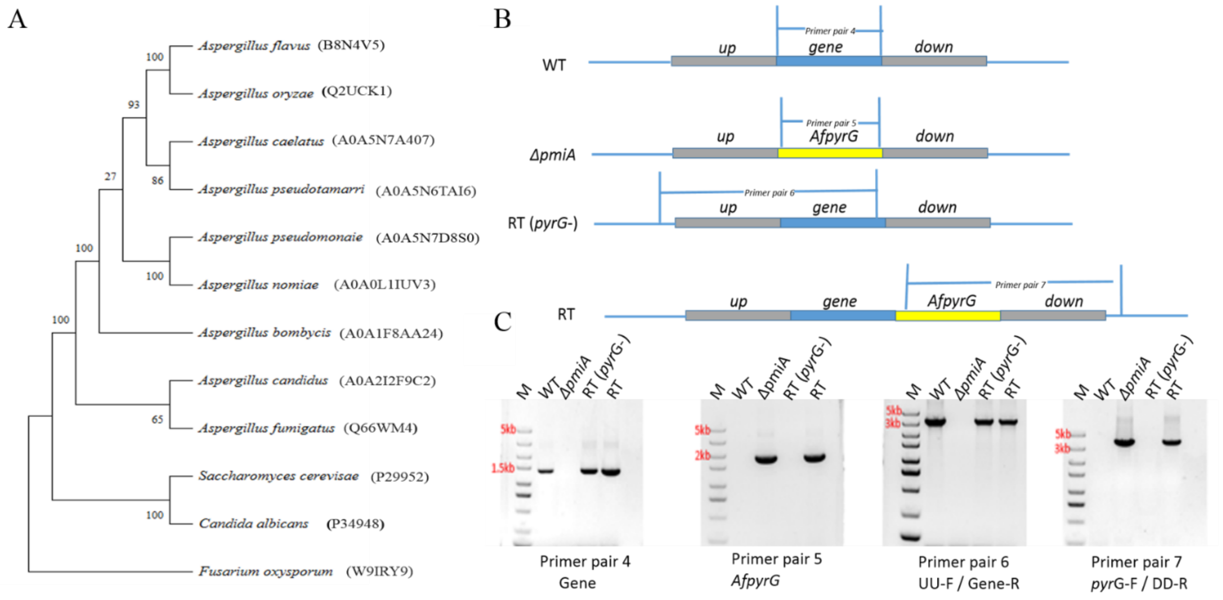

Fig. S1. A. Phylogenetic tree of PMI in *Aspergillus flavus* (B8N4V5) with other species**.** PMI sequences from *Aspergillus caelatus* **(**A0A5N7A407), *Aspergillus candidus* (A0A2I2F9C2), *Saccharomyces cerevisae* **(**P29952), *Aspergillus pseudotamarri* **(**A0A5N6TAI6)**,** *Aspergillus pseudomonaie* (A0A5N7D8S0), *Aspergillus bombycis* (A0A1F8AA24), *Aspergillus nomiae* (A0A0L1IUV3)**,** *Aspergillus oryzae* (Q2UCK1), *Aspergillus fumigatus* (Q66WM4), *Fusarium oxysporum* (W9IRY9)**,** and *Candida albicans* (P34948) were included and analyzed by MEGA 5. Neighbor joining method with a bootstrap value of 1,000 replicates was used to generate the phylogenetic tree. B, Strategies to generate the Δ*pmiA* and RT strains by homologous recombination. C, PCR analysis of the Δ*pmiA* and RT strains using four pairs of primers indicated in B. WT is wild type, Δ*pmiA* is the mutant, RT(*pyrG*$^-$) and RT are the revertant strains.

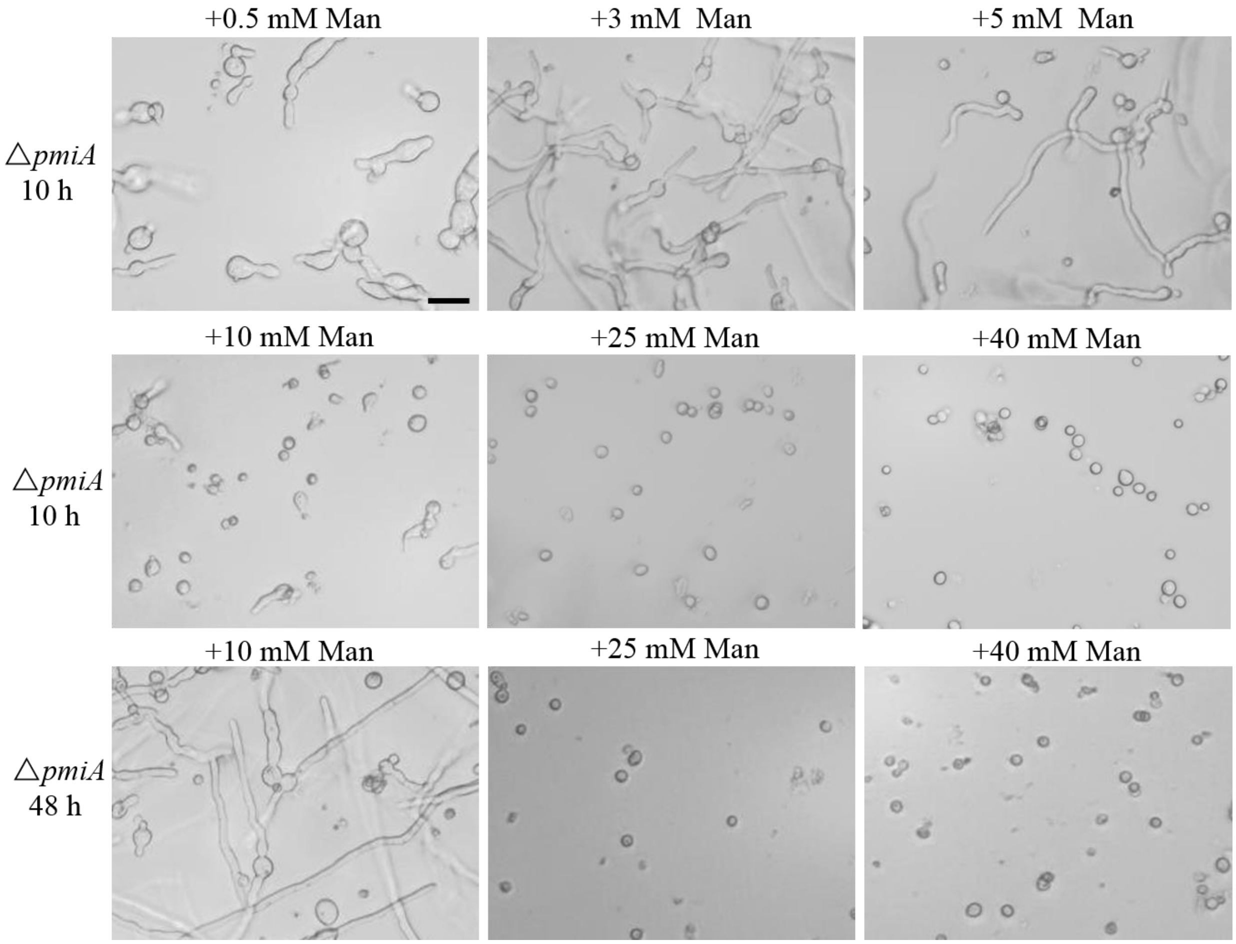

Fig. S2. **Germination morphology of the Δ*pmiA* mutant in liquid MMM.** DIC microscope (Leica) was used to record the mycelial morphology of Δ*pmiA* strain on MM liquid media supplemented with 0.5, 3, 5, 10, 25, and 40 mM mannose at 10 h and 48 h of cultivation at 37 ℃. Scale bar is 10 μm.

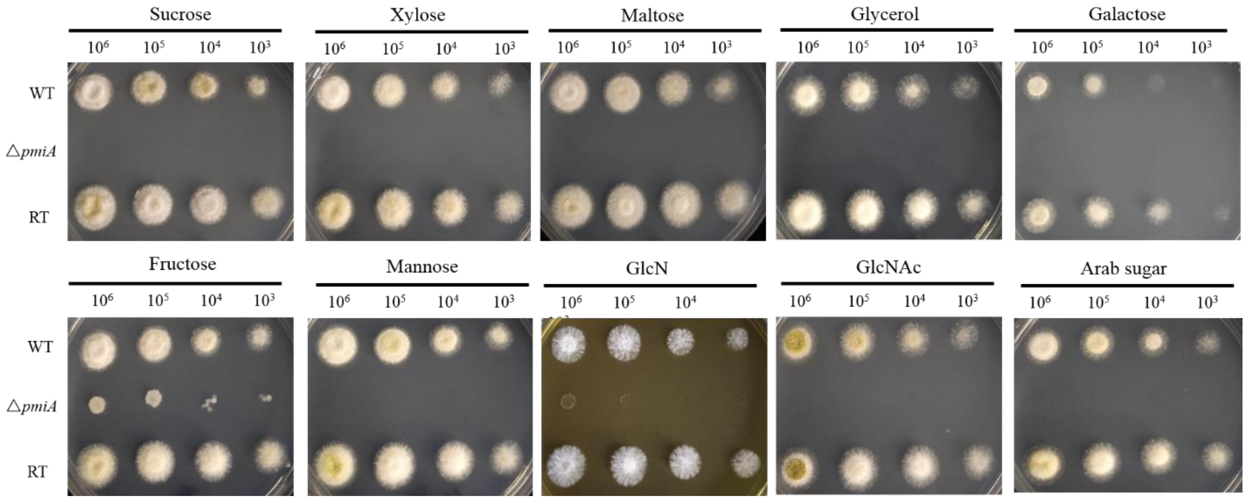

Fig. S3. Growth of Δ*pmiA* mutant on medium containing sucrose, xylose, maltose, glycerol, galactose, fructose, mannose, GlcN, N-acetylglucosamine (GlcNAc), and arabinose as sole carbon sources. Serially diluted conidia 10^6-10^3 of the WT, Δ*pmiA* and RT strains were point inoculated on the plates and incubated at 37 ℃ for 48 h.

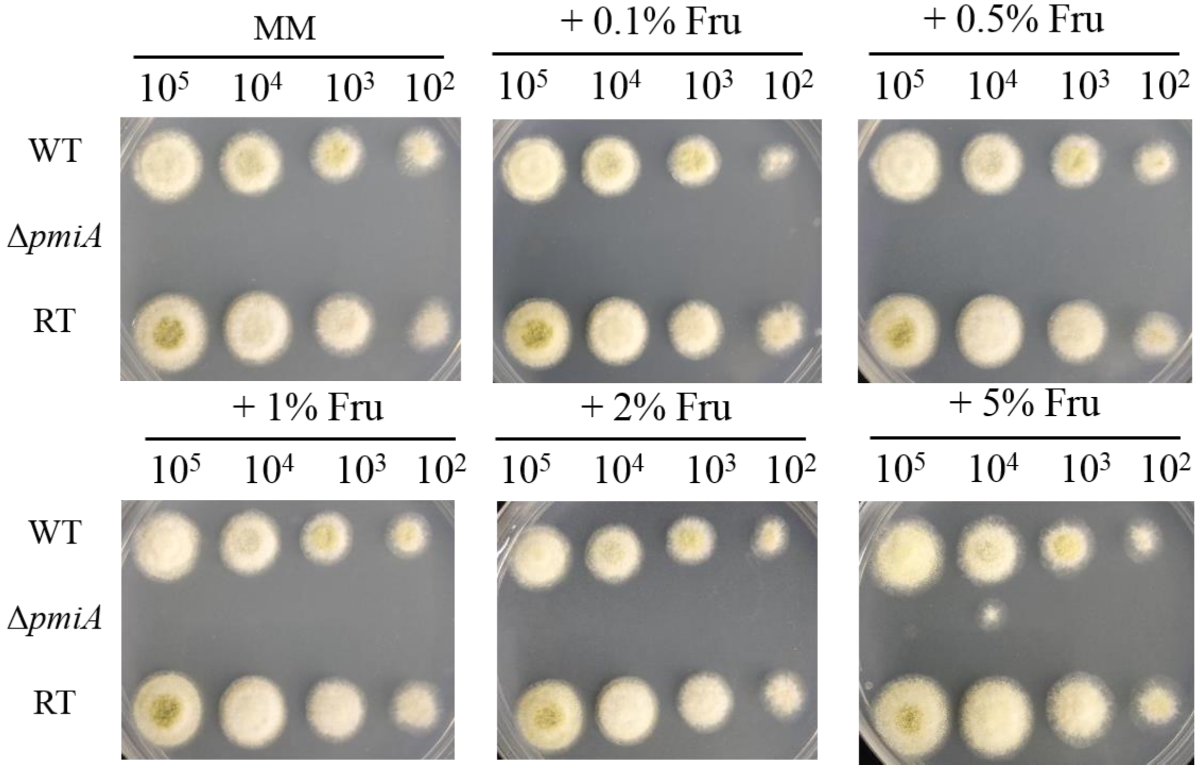

Fig. S4. Growth of PMI mutants at different concentrations of fructose (Fru). Freshly harvested conidia of the WT, Δ*pmiA* and RT strains were serially diluted (10^5-10^2) and point inoculated on MM media supplemented with 0.1%, 0.5%, 1%, 2% and 5% Fructose. Plates were incubated at 37 ℃ for 48 h.

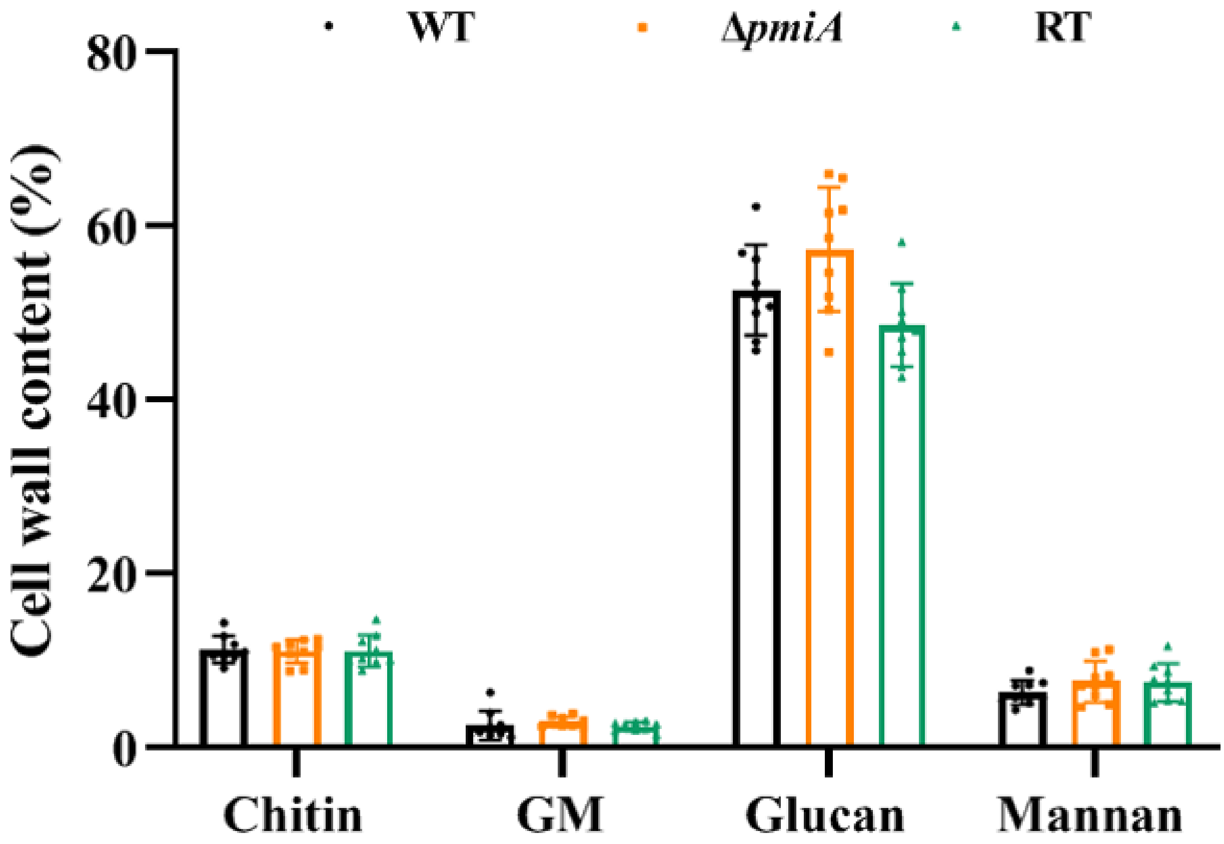

Fig. S5. Cell wall content analysis of Δ*pmiA* strain. 10^7 conidia of WT, Δ*pmiA* and RT strains were inoculated in MMM liquid medium and incubated at 37 °C for 48 hours. For the cell wall contents quantification dried mycelia (10 mg) were used. The experiment was conducted in three biological replicates.

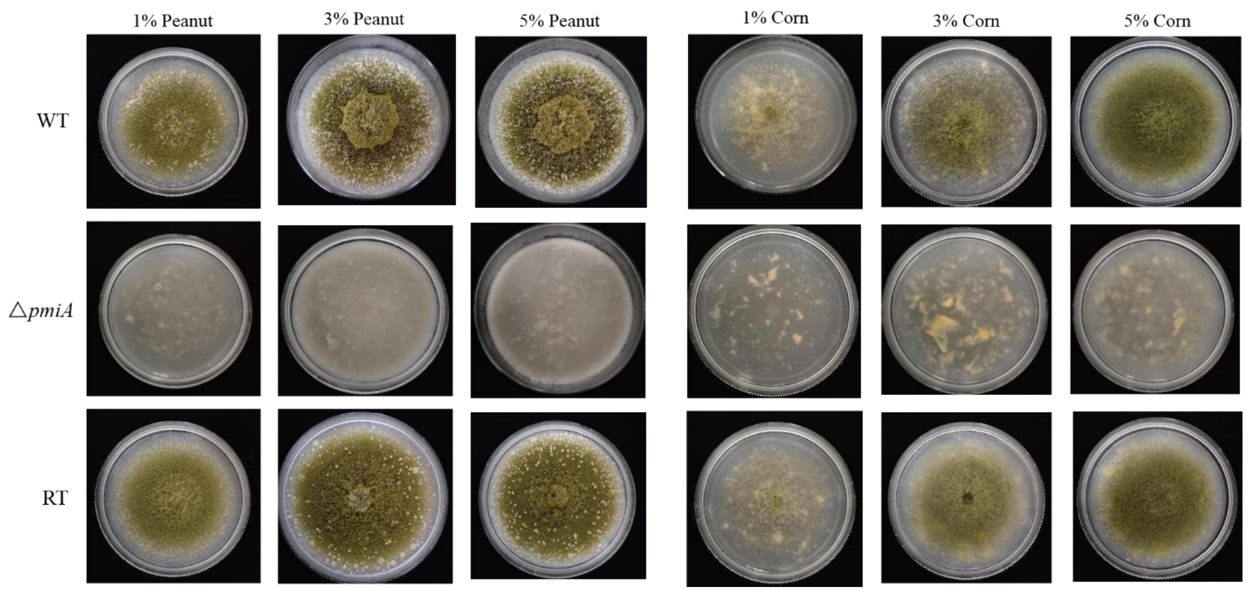

Fig. S6. Growth of the strains on plates supplemented with 1%, 3% and 5% of peanut and corn powder as nutrients. 10^5 fresh conidia of the WT, Δ*pmiA* and RT strains were inoculated in the center of the plates at 37 ℃ for 6 days.

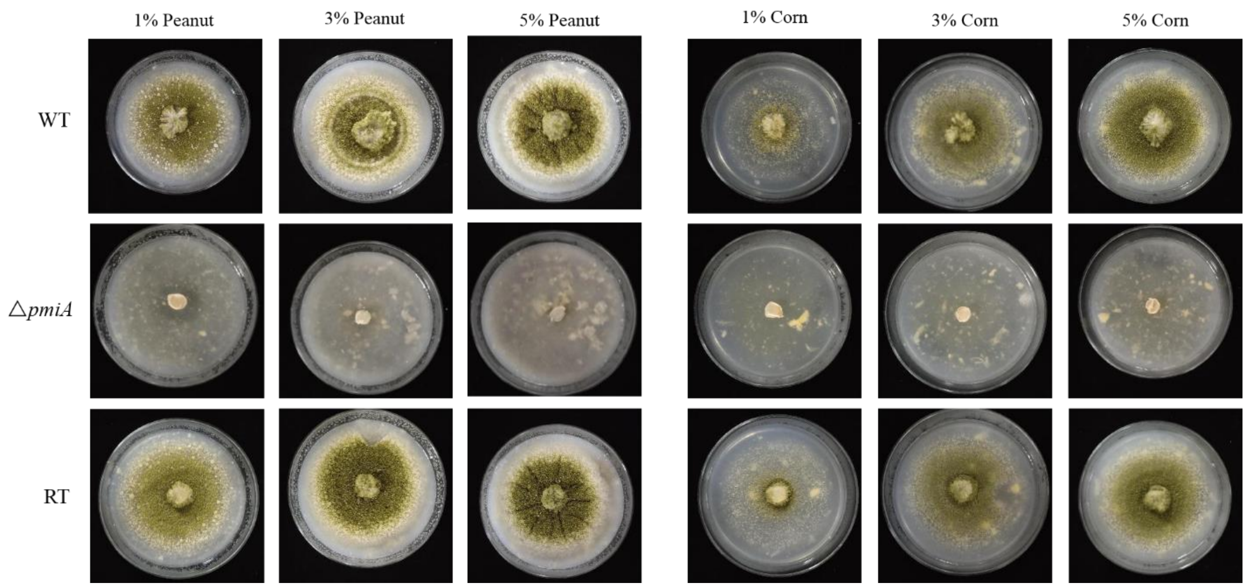

Fig. S7. Growth of the hyphae on plates supplemented with 1%, 3% and 5% of peanut and corn powder as nutrients. Hypha blocks of the WT, Δ*pmiA* and RT strains were inoculated in the center of the plates at 28 ℃ for 4 days.

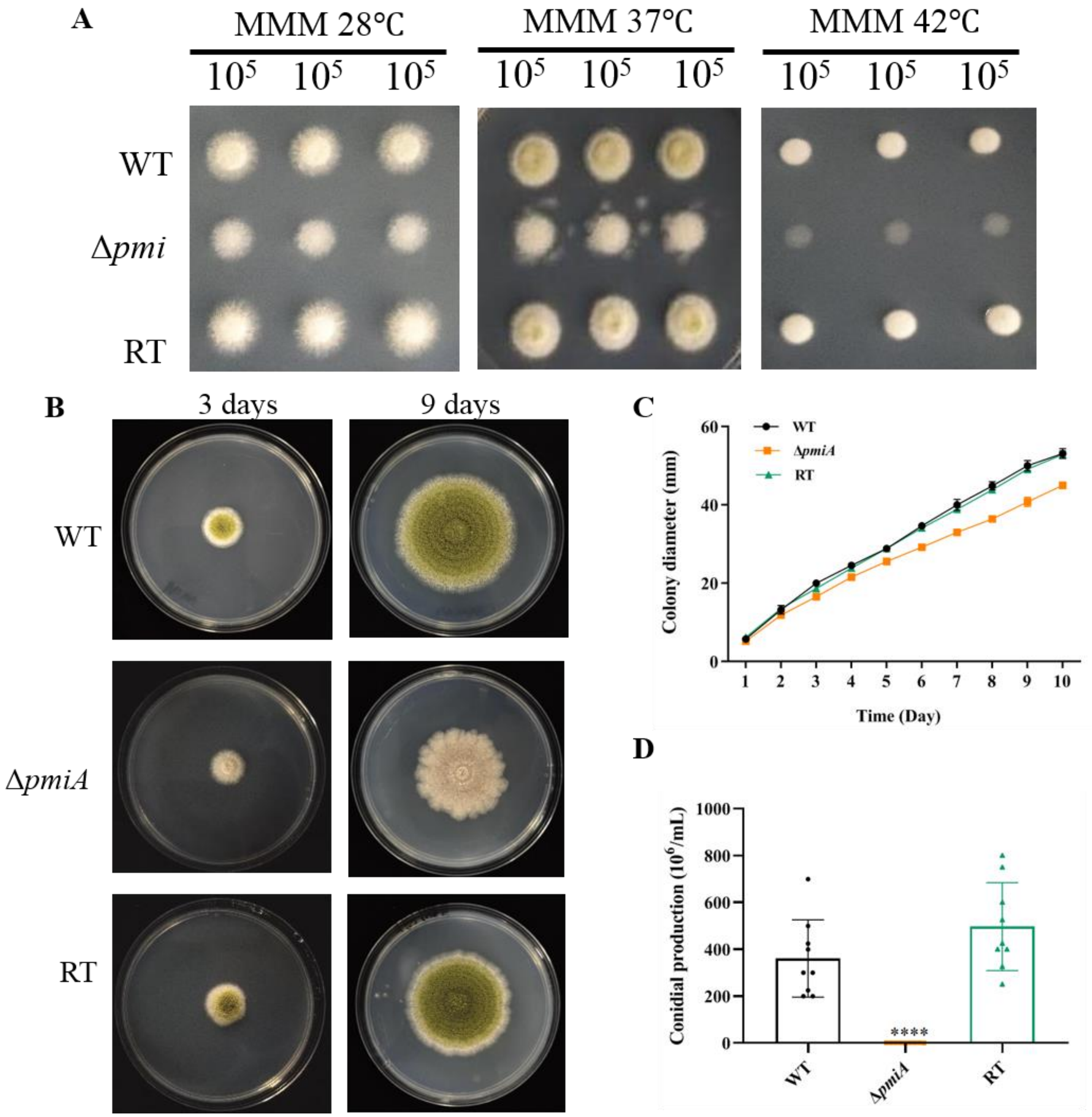

Fig. S8. Growth of the WT, Δ*pmiA* and RT strains on MMM. A, growth of WT, Δ*pmiA* and RT strains at different temperatures. B, conidia of the WT, Δ*pmiA* and RT strains were inoculated on MMM and incubated at 28 ℃ for 3 and 9 days. C, colony diameter was recorded daily for 10 days for the three strains grown on MMM plates. Three replicates were performed, and data were shown as mean ± *SD*. D, conidia production was measured using hemocytometer after 10 days of incubation on MMM at 28 ℃. Values represent the mean ± *SD*; multiple *t* test analysis was used to indicate statistical significance (****$p < 0.0001$).

Table S1. Primers used in the study

Primers	Primer sequences (5'-3')	Length (bp)
**Mutant construction**		
Afl PMI-U-F	aattcggatcttccagagatTGGTGCCTTCCGTCCCTT	1530
Afl PMI-U-R	gtttgaggcTTTGTAGAGCGTGACGTAACAGG	
Afl PMI-pyrG-F	cgctctacaaaGCCTCAAACAATGCTCTTCACC	1908
Afl PMI-pyrG-R	aatagtGTCTGAGAGGAGGCACTGATGC	
Afl PMI-D-F	gcctcctctcagacACTATTACTTAACAGCATTGAATGGATG	1534
Afl PMI-D-R	ttcaactgccgttcgacgatAAAAGAAAGGAAAGAAAGAATACATACCT	
**Mutant confirmation**		
Afl-PMI-G-U	AACCCTCCGACATCCTGAATC	1367
Afl-PMI-G-R	CATAGCCCTCCTTGACTTCCTC	
Afl-PMI pyrG -U	GCCTCAAACAATGCTCTTCACCC	1891
Afl-PMI-pyrG-R	GTCTGAGAGGAGGCACTGATGC	
Afl-PMI-UU-F	CCTTGTCATCTTCTTCGCCTTCA	3431
Afl-PMI-G-R	CATAGCCCTCCTTGACTTCCTC	
Afl-pyrG-F	GCCTCAAACAATGCTCTTCACC	3778
Afl-PMI-DD-R	CCGTTTGCTATCGTCCCAGTT	
**Revertant construction**		
2R Gene f	aattcggatcttccagagatATGCAGGTGCCTTTGCTTCG	1665
2R Gene R	ttgtttgaggcTTAGTGGCCGTTCACCATGTC	
*pyr*G f	ggccactaaGCCTCAAACAATGCTCTTCACC	1910
*pyr*G R	ggtttgtttcGTCTGAGAGGAGGCACTGATGC	
Down F	gcctcctctcagacACTATTACTTAACAGCATTGAATGGATG	1534
Down R	ttcaactgccgttcgacgatAAAGAAAGGAAAGAAAGAATACATACCTG	

Afl-PMI-U-F	CTTCCGTCCCTTCCTCCACTT	4529
Afl-PMI-D-R	TTCCTTCACTTTCGCATCCCT	

Table S2 Survival rate of the nematodes and *G. mellonella* larvae infected by the indicated strains.

Table S2A. Survival rate and hyphal filamentation rate of *glp-4*(bn2);*sek-1*(km4) worms infected by the indicated strains.

**Strain**	**Survival rate (%)**			**Hyphal filamentation at 24 h (%)**	**Worm numbers**
	**24 h**	**48 h**	**72 h**		
OP50	99 ±0.4	98 ±2.8	96 ±1.7	0	1552
WT	39 ±2.7	24 ±4.5	20 ±5.0	55 ±3.4	1814
Δ*PmiA*	94 ±6.3	89 ±6.7	79 ±13.4	0	1401
RT	45 ±17.5	27 ±8.9	19 ±8.6	51 ±17.2	1479

Three biological repeats (each with triplicates) were conducted and total counted worm numbers were indicated.

Table S2B. Survival rate of *G. mellonella* larvae infected with conidia of indicated strains.

Time	CK	WT	Δ*pmiA*	RT
24 h	93 ± 7	93 ± 3	99 ± 2	87 ± 6
48 h	80 ± 9	19 ± 7	78 ± 5	20 ± 10
72 h	80 ± 9	13 ± 2	73 ± 3	7 ± 3

Larvae treated with heat killed spores were used as control (CK). Three biological repeats (each with triplicates) were conducted.
